# Stomatal clustering in *Begonia* improves water use efficiency by modulating stomatal movement and leaf structure

**DOI:** 10.1002/pei3.10086

**Published:** 2022-07-04

**Authors:** Meng‐Ying Tsai, Chi Kuan, Zheng‐Lin Guo, Hsun‐An Yang, Kuo‐Fang Chung, Chin‐Min Kimmy Ho

**Affiliations:** ^1^ Institute of Plant and Microbial Biology Academia Sinica Taipei Taiwan; ^2^ Research Museum and Herbarium (HAST) Biodiversity Research Center, Academia Sinica Taipei Taiwan

**Keywords:** *Begonia*, stomata, stomatal clustering, stomatal movement, TOO MANY MOUTHS (TMM), water use efficiency (WUE)

## Abstract

Stomata are a pivotal adaptation of land plants and control gas exchange. While most plants present solitary stomata, some plant species experiencing chronic water deficiency display clustered stomata on their epidermis; for instance, limestone‐grown begonias. Moreover, the membrane receptor TOO MANY MOUTHS (TMM) plays a major role in spacing stomata on the epidermis in *Arabidopsis*, but the function of its *Begonia* orthologs is unknown. We used two Asian begonias, *Begonia formosana* (single stomata) and *B. hernandioides* (clustered stomata), to explore the physiological function of stomatal clustering. We also introduced the *Begonia TMM*s into *Arabidopsis tmm* mutants to study the function of *Begonia* TMMs. *B. hernandioides* showed higher water use efficiency under high light intensity, smaller stomata, and faster pore opening than *B. formosana*. The short distance between stomata in a cluster may facilitate cell‐to‐cell interactions to achieve synchronicity in stomatal movement. *Begonia* TMMs function similarly to *Arabidopsis* TMM to inhibit stomatal formation, although complementation by TMM from the clustered species was only partial. Stomatal clustering in begonias may represent a developmental strategy to build small and closer stomata to achieve fast responses to light which provides tight support between stomatal development and environmental adaption.

## Graphical abstract

Stomatal clustering is often observed in plants inhabiting arid or semi‐arid environments. However, the underlying physiological importance is unknown. Here, we used two genetically closely related Asian Begonia species as representatives for solitary and clustered types. We determined that stomatal clustering improves water use efficiency by facilitating stomatal dynamics. In addition to stomata, the associated leaf structure, including a multilayer (or multiseriate) epidermis, creates a large carbon dioxide sink for photosynthesis. Therefore, modulating stomatal development and leaf structure may improve plant fitness and potentially provide a means to increase biomass.

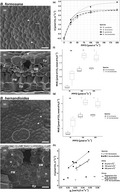

## 1 INTRODUCTION

Stomata are microscopic structures over the leaf epidermis that mediate gas exchange coming in and leaving the plant. These minute structures make plants an integral part of the global carbon and water cycles. Investigation of herbarium specimens collected over the last 200 years revealed a 40% decrease in stomatal density, likely a consequence of the rise in atmospheric carbon dioxide (CO_2_) concentrations since the beginning of the industrial revolution (Woodward, 1987). Rising CO_2_ concentrations might have also been associated with the observed increase in forest water use efficiency (WUE) due to the partial closure of stomata (de Boer et al., 2011; Keenan et al., 2013). Consequently, stomata not only bridge plants and climate but potentially modulate the global water cycle.

Atmospheric CO_2_ is the carbon source for plant photosynthesis. CO_2_ diffuses from the atmosphere into the substomatal cavity and then enters mesophyll cells and reaches the chloroplasts where carbon fixation takes place (Lawson & Blatt, 2014). In parallel, water evaporates from leaves in the reverse direction, generating a conundrum: Plants need to open their stomata to uptake carbon essential for photosynthesis but will lose water at the same time. Therefore, WUE reflects the rate of photosynthesis and transpiration, making it a useful metric that is often used to explore the trade‐offs between carbon gain and water loss through stomata.

Plants often cope with external stresses by reshaping their developmental programs to ensure survival and reproductive success in diverse environments. Stomatal development is one such program. Atmospheric CO_2_ concentrations and water availability are two factors at the interface between abiotic stimuli and stomatal development, resulting in the alternation of stomatal density in plants (Xu et al., 2016). In addition to the solitary form of dumbbell‐shaped stomata seen in monocots and the kidney‐shaped stomata of dicots, clustered stomata have been reported in 65 genera from 36 flowering plant families (Gan et al., 2010). Stomatal clustering has been suggested to be related to adaptation to specific environments. For example, the incidence of stomatal clustering in broad bean (*Vicia faba*) increases with the degree of water deficit and salinity (Gan et al., 2010; Hoover, 1986). Ecological studies of star begonia (*Begonia heracleifolia*) and lily pad begonia (*B. nelumbiifolia*), two Mexican begonias with clustered stomata, showed that plants inhabiting drier habitats tend to exhibit higher mean stomatal cluster size and a higher range of cluster sizes (Hoover, 1986). *Begonia plebeja*, an American species with two or more stomata clustered in one complex, showed higher WUE than *Begonia coccinea* (with solitary stomata) under low light intensity (~70 μmol·m^−2^·s^−1^) (Papanatsiou et al., 2017). In addition, a correlation between the occurrence of clustered stomata and a multiseriate epidermis has been previously reported (Boghdan & Barkley, 1972; Tang et al., 2002). These observations suggest that stomatal clustering and a multiseriate hypodermis assist with water conservation. However, what physiological function stomatal clusters hold and how they are formed are still unknown.

In most plants, stomatal distribution on the leaf surface obeys the one‐cell‐spacing rule, whereby a stomate is separated from another stomate by at least one pavement cell. In *Arabidopsis* (*Arabidopsis thaliana*), this cell‐to‐cell communication is mediated by a signaling pathway initiated by EPIDERMAL PATTERNING FAMILY (EPF) peptide ligands (Hara et al., 2007; Hara et al., 2009; Hunt & Gray, 2009) and the ERECTA family of receptors and its co‐receptor TOO MANY MOUTHS (TMM). *TMM* is a conserved and single‐copy gene in many plants including poplar (*Populus trichocarpa*), the grasses, and the moss *Physcomitrium* (*Physcomitrella*) *patens* (Peterson et al., 2010). Loss of TMM function results in contiguous stomatal clusters and increases the density of palisade mesophyll cells (Dow et al., 2017). Interestingly, *tmm* mutants have better WUE compared to wild‐type plants, suggesting that WUE is not only controlled by stomata but also by interlayer coordination between the epidermis and the mesophyll (Dow et al., 2017). Although TMM is well studied in *Arabidopsis*, its function in other plant species with clustered stomata remains poorly known.

Here, we selected two Asian *Begonia* species, *B. formosana* (with solitary stomata) and *B. hernandioides* (with clustered stomata), to study the relationship between stomatal morphology and WUE. *B. formosana* inhabits forest floors in Taiwan, the southern Ryukyus Islands, and Japan. *B. hernandioides* is found along the coastal limestone hills of northern Luzon and the Philippines. We examined intrinsic WUE (WUEi), leaf characteristics, and stomatal responses to determine the factors contributing to the different gas exchange strategies used in these two *Begonia* species. We also explored the function of TMM from *Begonia* species with different stomatal types to ascertain whether TMM participates in the formation of stomatal clustering in *Begonia*. Overall, our results showed that *Begonia* plants with clustered stomata have higher WUEi than *Begonia* plants with solitary stomata. This increase in WUEi was caused by the multiseriate epidermis and a rapid and synchronous stomatal response under saturated light conditions (100–200 μmol·m^−2^·s^−1^) in *B. hernandioides*. Moreover, complementation of the *Arabidopsis tmm* mutant was more effective when using the *TMM* locus from the solitary stomata *Begonia* species compared to *TMM* from the clustered stomata *Begonia* species in inhibiting stomatal formation in *Arabidopsis*.

## 2 MATERIALS AND METHODS

### 2.1 Plant material and growth conditions

Three biological replicates of *Begonia hernandioides* (with clustered stomata) and *Begonia formosana* (with solitary stomata) were grown in the natural light greenhouse of the Biodiversity Research Center at Academia Sinica (BRCAS), Taiwan, with the maximum light intensity, maintained about 100 μmol·m^−2^·s^−1^. *B. hernandioides* was collected from the Philippines by the collector Rosario Rubite (#106), and *B. formosana* was collected from Hsiulin, Hualien, Taiwan, by the collector C.K. Yang (#1637).

### 2.2 Cryo‐SEM observations of the leaf abaxial epidermis and leaf cross sections

Mature leaves were collected from the second or third node of the two *Begonia* spp. The major veins were removed to retain only the inter‐vein leaf blade, which was dissected into 5‐ to 10‐mm^2^ sections. Prepared leaf sections were attached on a stub vertically or horizontally with Tissue‐Tek O.C.T. compound mixed with carbon powder and then cryo‐fixed by liquid nitrogen directly, before being transferred to a pre‐cooled cryo‐preparation chamber for sample preparation. After etching for 15 min and platinum coating for ca.1–2 min, the samples were transferred to a pre‐cooled scanning electron microscope (SEM). For observing leaf cross sections, the cryo‐fixed leaf samples in the cryo‐preparation chamber were fragmented with a razor knife after etching. All samples were observed at 20 kV. The digital images of the stomatal morphology from the abaxial epidermis and each leaf cross section from the two *Begonia* spp. were processed and labeled by Adobe Illustrator software.

### 2.3 Gas exchange measurements

Measurements of gas exchange were conducted using the LI‐COR 6400XT Infrared Gas Analyzer with a 6‐cm^2^ standard leaf chamber. The environment of the leaf chamber was maintained at 25°C leaf temperature, 1.0–1.2 vapor water deficit, and 400 ppm CO_2_. Gas exchange responses were measured at 50, 100, and 200 μmol·m^−2^·s^−1^ using an external light source (LI‐COR 6400–18). The parameter of stomatal ratio was set to zero, as stomata are only on the abaxial leaf epidermis of *Begonia* spp. All plants were moved from the greenhouse to the laboratory for environmental acclimation the day before the measurement (ca. 12 h). To find the suitable saturated light for most *Begonia* species in the same greenhouse, we used at least two individuals of *B. cavaleriei* and *B. quixiensis* to construct light curves for Asia‐begonias. Light intensities at 50, 100, and 200 μmol·m^−2^·s^−1^ were then applied to the experiments of *B. hernandioides* and *B. formosana*. To reduce the fluctuation of gas‐exchange response in experimental plants, ca. 70 μmol·m^−2^·s^−1^ artificial light in a laboratory was used for 1 h before the gas‐exchange measurement. Those two *Begonia* species showed the same diurnal pattern; hence, we made the gas‐exchange measurement of each individual plant starting from 8 am to 3 pm throughout the day. One individual was measured per day. Three plants of *B. formosana* and *B. hernandioides* were measured on different days.

### 2.4 Leaf morphology and chlorophyll contents

Leaf mass per area (LMA) was calculated as the ratio between leaf dry mass (g) and leaf area (m^2^). Leaf thickness (mm) was measured with a vernier scale. Chlorophyll contents were measured with a chlorophyll content meter (CCM‐300, Opti‐Sciences, Inc, USA).

### 2.5 Quantification of stomatal images and calculation of g_s max_

Stomatal patterns in *B. formosana* and *B. hernandioides* from nail‐polish peels of abaxial epidermis were observed under a Leica light microscope (DM 500) and recorded by a Canon digital camera (EOS 800D). The stomatal density (SD), stomatal index (SI), and the width and length of stomata and guard cells were then quantified with Fiji/ImageJ (Schindelin et al., 2012). Because it is difficult to determine the open or closed state of stomata based on nail‐polish peels, the maximum pore of a stomate (*a*_*max*_) was estimated to be an ellipse with the major axis being stomatal length and the minor axis being half of the stomatal length. The stomatal depth (*l*) was estimated as the half‐width of guard cells. The *g*_*s max*_ was calculated based on the following equation from Dow et al. (2014):

$\text{anatomical}\;g_{s\cdot \max}=\left(d\times \mathrm{SD}\times a_{\max}\right)/\left(v\cdot \left(l+\pi /2\right)\right),$

where *d* (m^2^·s^−1^) is the diffusivity of water in air and *v* (m^3^·mol^−1^) is the molar volume of air in 25°C.

### 2.6 Response of stomatal opening

The day before the experiment, plants were transferred to constant darkness for at least 12 h to induce stomatal closure. Mature leaves from the second or third node position were collected, the major vein was removed, and the leaf blade was dissected into 1‐cm^2^ squares and submerged into the opening buffer. To capture images from the same position of the leaf section, the microscope light source was used at a light intensity maintained to 100–200 μmol·m^−2^·s^−1^ using a light meter (LI‐250A, LI‐COR) with a quantum sensor (LI‐190, LI‐COR). Leaf sections were observed under a Leica light microscope (DM 500). All images were recorded every 30 for 240 min (nine images taken per sample). The preparation of the opening buffer followed the method of Papanatsiou et al. (2017) with a few modifications, including 10 mM MES and 50 mM KCl. After testing for series of pH adjustments from 4.0 to 9.0, alkaline pH offered better responses for stomatal opening in the two *Begonia* spp. tested here. The opening buffer was thus adjusted to a pH of 8.86. All image recordings and quantification were performed as above for stomatal images for *g*_*s max*_.

### 2.7 Generation of transgenic constructs

*Begonia TMM* genes were amplified by PCR from genomic DNA extracted from *B. formosana* and *B. hernandioides* (F primer: 5'‐GCC GCG GCC ACC ATG TTT ATT CAC CTA CTT ATT TCC CC‐3'; R primer: 5'‐GCT GGC GCG CCC GCA ACC CAA CTT AAG TGT AAA CCA CA‐3') and using restriction enzyme cloning into the pENTR/D‐TOPO vector (Thermo Fisher). An LR Clonase™ reaction was used to recombine the resulting subcloned *BlTMM* and *BhTMM* coding sequences into the R4pGWB540 vector to be placed under the control of the cloned *AtTMM* promoter (Nakagawa et al., 2008). The binary constructs were then introduced into *Agrobacterium tumefaciens* GV3101 and used for *Arabidopsis* floral dip transformation. Plants from the T2 generation were used for the data collection of stomatal density.

### 2.8 Data statistical analyses

All statistical analyses were performed in *R version 4.0.3*. Graphs were produced using the *ggplot2* package in R. All data were first subjected to the Shapiro–Wilk test to check for normality and to the equal variance test to check homogeneity of variance. If the data were deemed to be normally distributed with homogenous variance, a Student's *t*‐test, or a Mann–Whitney rank‐sum test was conducted for two‐sample comparisons; a Kruskal‐Wallis one‐way analysis of variance (ANOVA) on ranks was conducted for comparing multiple samples. The gas exchange data, such as *A*, *E*, and *g*_*s*_ parameters between different light intensities in the same species, were compared by one‐way RM ANOVA. The association of stomatal opening patterns within species or individuals was tested by correlation analysis with Spearman's rank correlation coefficient. The relationship between distance and response of stomatal opening was tested by linear regression analysis. The α‐level was set to 0.05.

## 3 RESULTS

### 3.1 *B. hernandioides* leaves have large substomatal cavities and a multiseriate hypodermis

**FIGURE 1:**
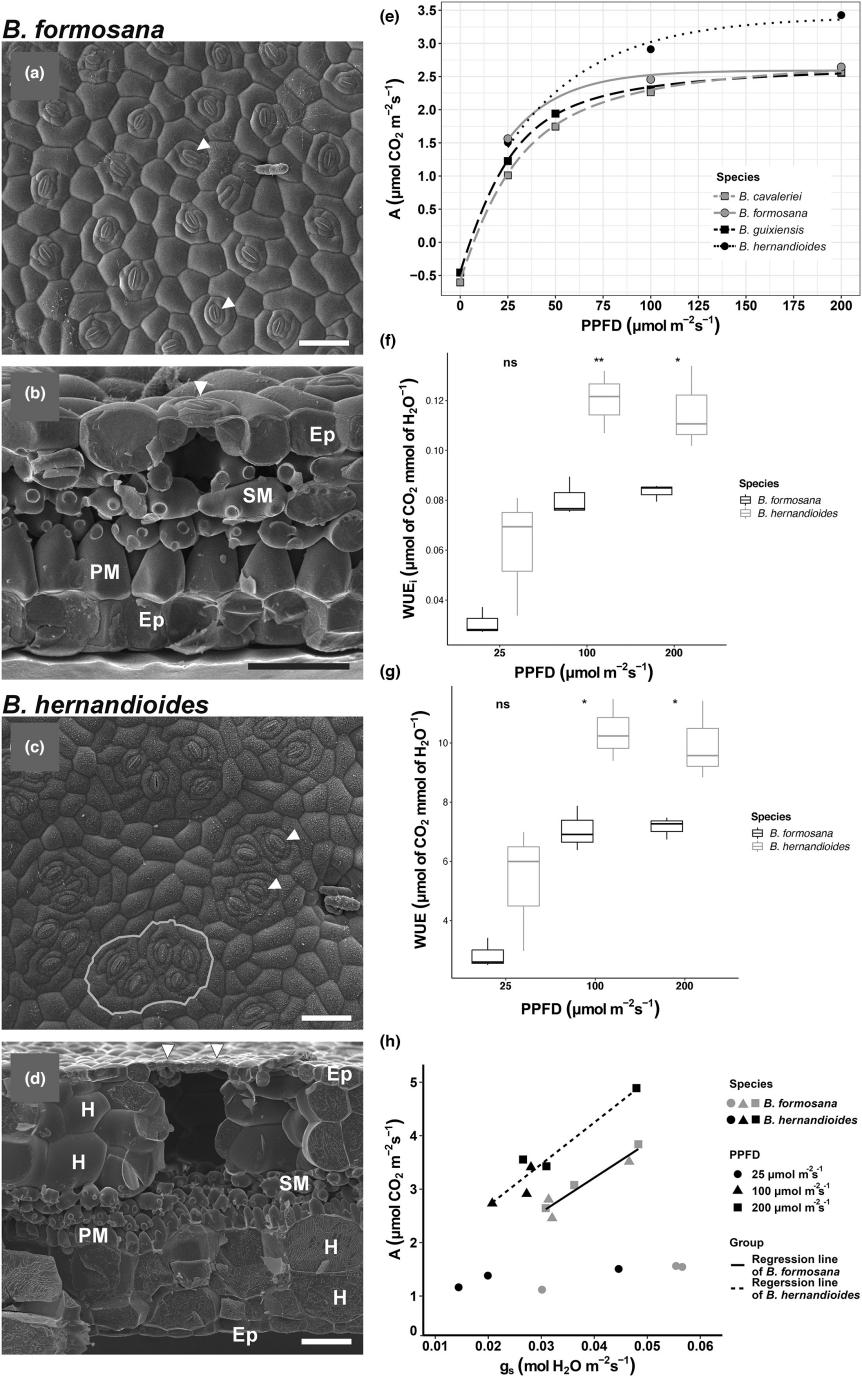
Scanning electron microscopy (SEM) images from the leaves of two *Begonia* species and their water use efficiency under well‐watered growth conditions. (a and c) Abaxial view of *B. formosana* (solitary stomata) (a) and *B. hernandioides* (clustered stomata) (c). Arrowheads indicate guard cells. Five stomata packed in a single cluster are circled in (c). (b and d) Cross‐section view of leaf layers and the substomatal cavity below a stomate (arrowhead) in *B. formosana* (b) and *B. hernandioides* (d). *B. hernandioides* presents a large substomatal cavity with multiseriate hypodermis. Scale bars, 100 μm. Ep, epidermis. H, hypodermis. PM, palisade mesophyll. SM, spongy mesophyll. (e) Changes in net CO_2_ assimilation rate (a) with photosynthetic photon flux density (PPFD) across four *Begonia* species: *B. cavaleriei* and *B. formosana* (solitary), and *B. guixiensis* and *B. hernandioides* (clustered). (f and g) Intrinsic WUE (WUEi) (f) and instantaneous WUE (WUE) (g) under three light intensities. Asterisks indicate significant differences between the two *Begonia* species, as determined by Student's *t*‐test (*n* = 3 biological replicates, **p < .01 and *p < .05). (h) Relationship between net CO_2_ assimilation rate (a) and stomatal conductance (gs) in the two *Begonia* species. Regression lines were obtained from measurements at 100 and 200 μmol m^−2^ s^−1^in *B. formosana* (solid line) and *B. hernandioides* (dashed line) (*n* = 3 plants).

We characterized the leaf morphology of two *Begonia* species by cryo‐scanning electron microscopy (Cryo‐SEM) (Figure 1). The abaxial epidermis of *B. formosana* presented solitary stomata (Figure 1a, arrowheads), while that of *B. hernandioides* displayed four to six stomata grouped into a cluster (Figure 1c, white circle). Cross‐section images indicated that *B. formosana* has thinner leaves and smaller substomatal cavities than *B. hernandioides* (Figure 1b and d). In *B. hernandioides*, stomata of the same cluster (Figure 1d, arrowheads) shared the same substomatal cavity (Figure 1d). In addition, *B. hernandioides* leaves showed a multiseriate hypodermis under both epidermis and resulted in a large substomatal cavity (Figure 1d).

### 3.2 *B. hernandioides* has higher WUEi under saturated light conditions

To explore the physiological significance of stomatal clustering, we selected four Asian *Begonia* species with different stomatal morphologies; *B. cavaleriei* and *B. formosana* (solitary stomata), *B. guixiensis* (one to three stomata in a cluster), and *B. hernandioides* (more than four stomata in a cluster). We measured net CO_2_ assimilation rates (*A*) in response to varying light intensity (Figure 1e). All four *Begonia* species exhibited similar photosynthetic responses, as *A* rose exponentially from 25 μmol·m^−2^·s^−1^, reaching a plateau around 100 μmol·m^−2^·s^−1^, with little change in *A* when increasing light intensity to 200 μmol·m^−2^·s^−1^ (Figure 1e). Based on this result, we focused on the three light irradiance levels 25 μmol·m^−2^·s^−1^ (exponential point), 100 μmol·m^−2^·s^−1^ (beginning of light saturation), and 200 μmol·m^−2^·s^−1^ (beyond light saturation) to study WUE. We selected the two *Begonia* species *B. formosana* and *B. hernandioides* for their similar leaf shapes and leaf sizes but contrasting stomatal types.

WUE can be expressed as intrinsic WUE (WUEi), via net carbon assimilation rate (*A*, μmol·m^−2^ ·s^−1^) and stomatal conductance (*g*_*s*_), or as instantaneous WUE via *A* and transpiration rate (*E*, mol·H_2_O m^−2^·s^−1^). We thus measured net CO_2_ assimilation rate (*A*), stomatal conductance (*g*_*s*_), and transpiration rate (*E*) in *B. formosana* and *B. hernandioides* under the same well‐watered growth conditions and at three light intensities (Table S1). We observed no significant differences in various parameters of gas exchange (*A*, *g*_*s*_, or *E*) between the two *Begonia* species (Table S1). However, WUEi and WUE of *B. hernandioides* did increase compared to *B. formosana* under 100 and 200 μmol·m^−2^·s^−1^ significantly (Figure 1f and g, *p* < .05, Student's *t* test).

To determine which component contributed to higher WUEi in *B. hernandioides*, we plotted *A* as a function of *g*_*s*_ under the three light intensities (Figure 1h). When excluding data points collected at 25 μmol·m^−2^·s^−1^, *A* and *g*_*s*_ followed a linear relationship with the same slope in both species, indicating that the strategy employed by *B. hernandioides* to maintain higher WUEi comes from a combination of slightly higher *A* and slightly lower *g*_*s*_ than in *B. formosana*.

### 3.3 Leaf structures and stomatal morphology determine WUEi in *B. hernandioides*

The photosynthetic potential of a leaf (*A*) has been reported to correlate with internal leaf characters such as chlorophyll contents, leaf mass per area (LMA, g·m^−2^), and leaf thickness (mm) (Buttery & Buzzell, 1977; Flexas et al., 2008; Hanba et al., 1999; Henry et al., 2019; Papanatsiou et al., 2017). The anatomical maximum rate of stomatal conductance to CO_2_ (anatomical *g*_*s max*_ or theoretical *g*_*s*_) contributed by stomatal pattern (as measured by stomatal density; SD) and stomatal structure (theoretical maximum stomatal pore, *a*_*max*_) connects stomatal development and leaf photosynthetic potential (Dow et al., 2014; Dow et al., 2017). Therefore, understanding the above parameters should help us understand which component(s) plays a major role in the higher WUEi seen in clustered *Begonia*.

We detected no significant differences in chlorophyll contents, SD, or *a*_*max*_ between the two *Begonia* species (Tables 1 and 2). However, *B. hernandioides* did have lower anatomical *g*_*s max*_, indicating that the lower *g*_*s*_ seen in *B. hernandioides* results from the combination of stomatal structure (*a*_*max*_) and stomatal pattern (SD). *B. hernandioides* also had higher LMA and leaf thickness (Tables 1 and 2), suggesting that higher WUEi is also influenced by leaf structure.

**TABLE 1:** Summary of leaf traits for *B. formosana* and *B. hernandioides*

Parameters	*B. formosana*	*B. hernandioides*	*p*‐value^a^
Chlorophyll content (mg·m^−2^)	1303.3 ± 15.9	1309.2 ± 44.5	.840
Leaf thickness (mm)	0.36 ± 0.02	0.55 ± 0.01	**<.001**
LMA (g·m^−2^)	17.33 ± 0.63	28.06 ± 1.53	**.011**

^a^ Student's *t*‐test from *n* = 3 biological replicates.

**TABLE 2:** Summary of stomatal traits in *B. formosana* and *B. hernandioides*

Parameters	Species		*p*‐value^a^
	*B. formosana*	*B. hernandioides*	
Stomatal density (μm^−2^)	67.83 ± 16.78	51.23 ± 8.66	.400
Stomatal size (μm^2^)	568.6 ± 140.1	519.4 ± 82.6	**.037**
*a*_*max*_ (μm^2^)	193.4 ± 85.9	183.7 ± 52.6	.921
Anatomical *g*_*s max*_ (mol m^−2^ s^−1^)	0.07 ± 0.01	0.06 ± 0.00	**<.001**

^a^ For each species, 90 stomata were measured (*n* = 3 biological replicates). Comparison of stomatal density and other stomatal parameters between the two *Begonia* species was conducted by Mann–Whitney rank‐sum test. Significant differences are in bold.

Interestingly, *B. hernandioides* formed smaller stomata (*p* = .037, Mann–Whitney rank‐sum test, Table 2) compared to *B. formosana*. Smaller stomata are thought to respond faster than larger stomata (Drake et al., 2013; Kardiman & Ræbild, 2017). Therefore, the higher WUEi of *B. hernandioides* may combine changes in leaf structure, stomatal morphology, and quicker stomatal response.

### 3.4 Fast stomatal opening of clustered stomata

**FIGURE 2:**
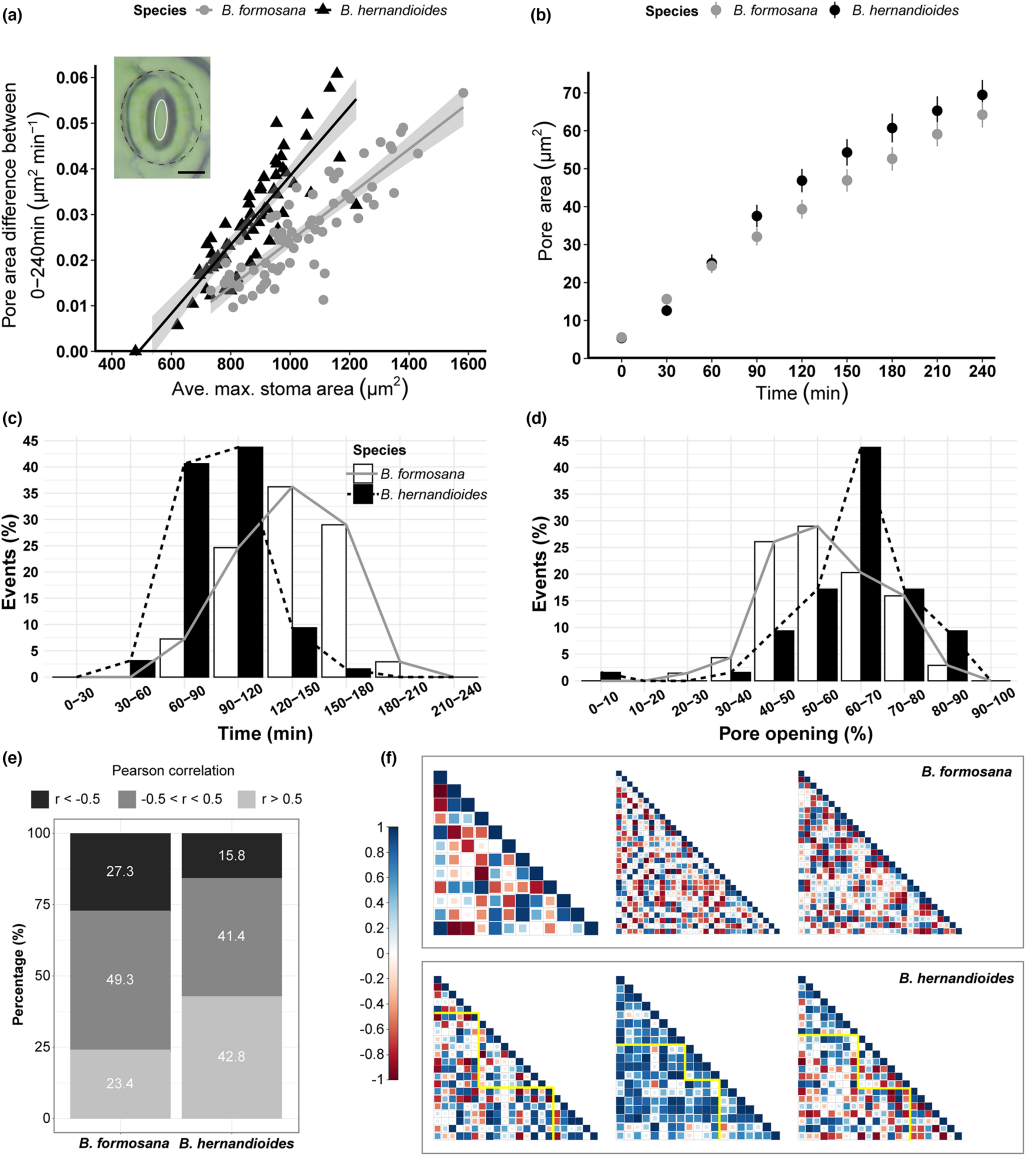
Responses and patterns of pore opening in the two *Begonia* species *B. hernandioides* and *B. formosana*. (a) Relationship between the rate of pore opening and the average maximum stomatal area over 240 min. *B. hernandioides*, black line and symbols; *B. formosana*, gray line and symbols. The null hypothesis of equal slopes between the two regression lines was rejected by ANOVA with the *p* < .05. Inset, Pore area (solid white line) and stomatal area (dashed black line). Scale bar, 10 μm. Maximum stomatal area was calculated using the average of stomatal area of each stomate from the last two time points. (b) Pore area increases over time. *n* = 69 stomata (*B. formosana*), *n* = 64 stomata (*B. hernandioides*). Data are shown as means ± standard error (S.E.). (c) Distribution of the time required to reach half‐pore opening (*A* ^*1/2*^). *B. formosana*, solid line; *B. hernandioides*, dashed line. (d) Distribution of the percentage of open pores 2 h into the experiment (*t* ^*1/2*^). Most pores in *B. hernandioides* were 60–70% open at 2 h. *B. formosana*, solid line; *B. hernandioides*, dashed line. (e) Pearson's correlation coefficients (*r*) for pairwise comparisons between individual opening dynamics from 30 to 150 min in *B. formosana* and *B. hernandioides*. *B. hernandioides* shows more positive correlations than *B. formosana*. (f) Heatmap representation of Pearson's correlation coefficients (*r*) between 30 and 150 min for *B. formosana* and *B. hernandioides* in each sample (*N* = 3). Stomata with similar pore dynamics cluster together and exhibit more intense colors. Blue and red represent positive and negative correlations, respectively. *n* = 69 stomata (*B. formosana*), *n* = 64 stomata (*B. hernandioides*). Different clusters in *B. hernandioides* were separated by yellow line.

Next, we measured the stomatal response of the two *Begonia* species. Accordingly, we first placed whole plants in complete darkness for 12 h to close their stomata. Just before the measurement, we irradiated leaves (100–200 μmol·m^−2^·s^−1^) in opening buffer and recorded pore size every 30 min (Figure 2a and Figure S1). We quantified pore area and stomatal size (white solid line and black dashed line, respectively; Figure 2a, inset, using an elliptic approximation using ImageJ/Fiji). The gradual increase in pore size over time indicated that the opening buffer is effective in both *Begonia* species (Figure S1a and b). To remove possible artifacts arising from incomplete stomatal closure at the starting point and variable stomatal sizes between the two *Begonia* species, we normalized the data by subtracting the initial pore size (0 min) over the entire time course (zeroing, Figure S1c and d) or zeroed and normalized pore size by the average maximum length of individual stomata (Figure S1e and f). However, the resulting graphs exhibited similar trends as non‐normalized data (Figure S1a and b). Therefore, we used the original data for the following analyses.

In both *Begonia* species, the rate of pore opening was positively correlated with stomatal size (Figure 2a). The steeper slope in *B. hernandioides* compared to *B. formosana* (Figure 2a) suggested that *B. hernandioides* stomata open faster than those of *B. formosana* for the same stomatal size (*p* < .001, linear regression model and ANOVA to compare slopes). When the average pore area was plotted over time, we noticed that *B. formosana* showed greater stomatal opening 30 min into the time course, while *B. hernandioides* started to present bigger pore sizes than *B. formosana* after 90 min (Figure 2b). The difference in stomatal opening between *B. formosana* and *B. hernandioides* was statistically significant between 30 and 90 min (*p* = .002, Mann–Whitney rank‐sum test). Thus, the smaller stomata of *B. hernandioides* exhibited faster opening kinetics.

### 3.5 Synergistic effect of pore opening in clustered stomata

Since cluster stomata appeared to open faster, we wondered whether individual stomata within clusters might affect its neighbors, which would lead to a similar stomatal response across neighboring stomata. We estimated this effect with a half‐stomatal opening (*A* ^*1/2*^), that is, the time required to reach half of the final pore size, and the extent of stomatal opening 2 h into the experimental time course (Figure 2c and d). For comparison, we set the percentage of pore opening to 0 at the starting point (0 min) and to 100% at the endpoint (240 min) (Figure 2d). The distribution of *A* ^*1/2*^ in *B. hernandioides* was more right‐skewed than that in *B. formosana* (Figure 2c). After 2 h, the median pore opening in *B. hernandioides* had reached 60–70% opening compared to 50–60% in *B. formosana* (Figure 2d). These results suggested a quicker stomatal response in *B. hernandioides*, which was consistent with the above observations (Figure 2a). Interestingly, *B. hernandioides* showed a narrower distribution in pore opening compared to the broader peak of *B. formosana* at 2 h (Figure 2d). We observed a similar trend in the *A* ^*1/2*^ distribution (Figure 2c). Taken together, *B. hernandioides* exhibited a faster and more synchronous pore opening than in *B. formosana*.

To further investigate synchronicity across leaf stomata, we measured the Pearson's correlation coefficient (*r*) to estimate the relationship between opening patterns of individual stomata between 30 and 150 min. We obtained more positive (*r* > .5) correlations in *B. hernandioides* than in *B. formosana* (Figure 2e). When we visualized the correlations as a heatmap of the correlation matrix in the individual samples, *B. hernandioides* exhibited larger intense blue zones relative to *B. formosana* (Figure 2f). Since the middle sample in *B. hernandioides* showed higher correlations compared to the other two samples (Figure 2f), we excluded it and calculated Pearson's correlation coefficient (*r*) again. The result showed there were still more positive correlations in *B. hernandioides* than in *B. formosana* (Figure S1g), indicating that clustered stomata tend to behave more similarly than solitary ones.

### 3.6 The rate of pore opening weakly correlates with mean distance between stomata in a cluster

**FIGURE 3:**
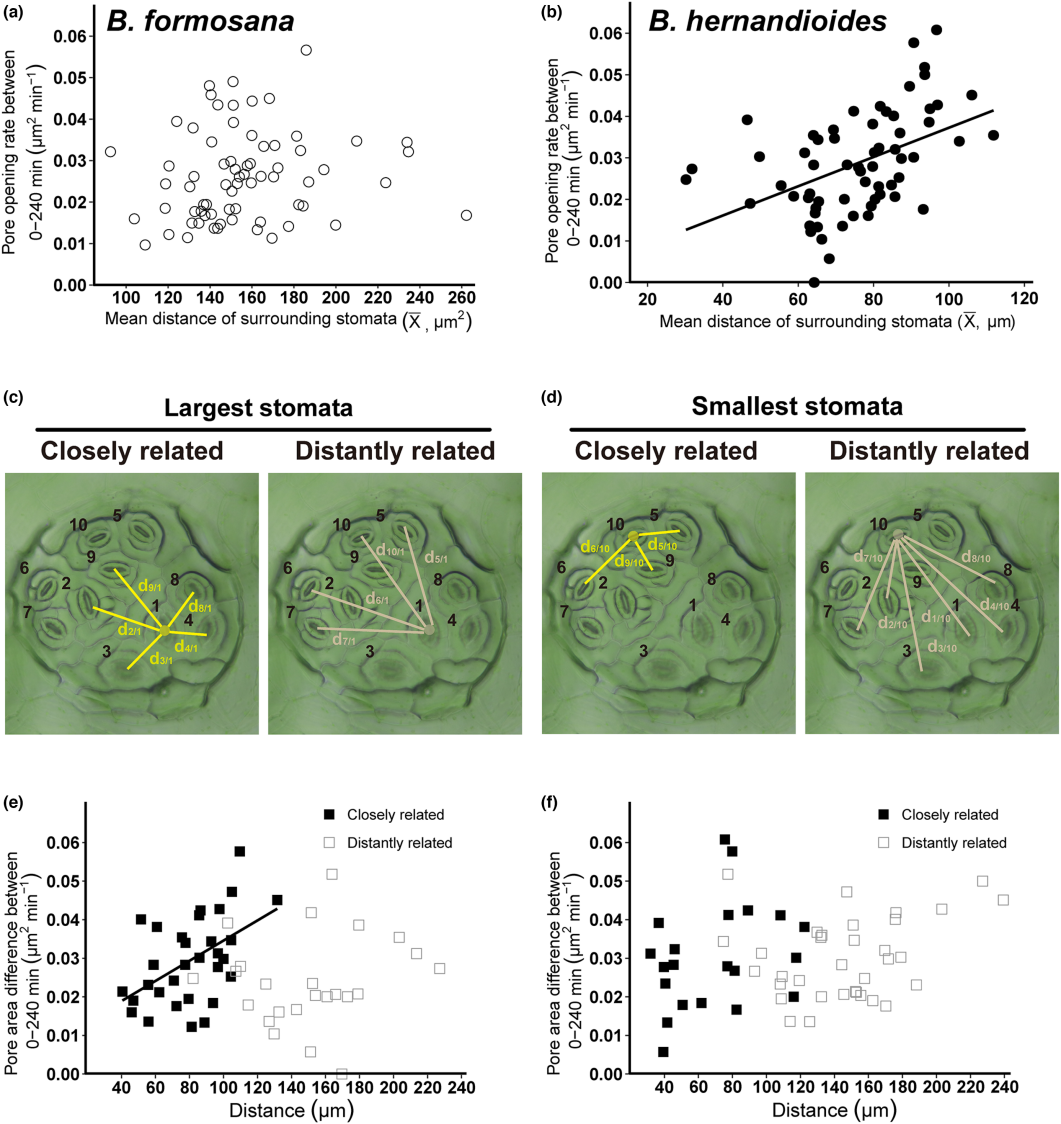
Relationship between opening rate and distances between stomata. (a and b) Influence of mean stomatal distance on pore opening in *B. formosana* (a) and *B. hernandioides* (b). There was no relationship between pore opening and mean distance (X̅) in *B. formosana* (*n* = 69, *p* = .265, Student's *t*‐test). There was a weak but significant relationship in *B. hernandioides* (*n* = 64, *r* ^2^ = .22, *p* < .001, *t*‐test). (X̅) is the average distance between one stomate and its closely related neighbor stomata. (c and d) Illustration of closely related and distantly related stomatal connection in *B. hernandioides*. In contrast to panel (b), clustered stomata were ranked here by their final pore area, with #1 being the largest and #10 the smallest. (e and f) To see the influence of a stomatal size on the opening of its neighbors, the pore dynamics and the distances between the largest (c) or smallest (d) stomate to its adjacent or faraway neighbors are shown. (e) Stomata closely related to the largest stomate show a positive correlation (solid dots, *n* = 31, *r* ^2^ = .25, *p* = .004, Student's *t*‐test), but distantly related stomata do not (open dots, *n* = 24, *p* = .538, Student's *t*‐test). (f) Stomata surrounding the smallest stomate do not show a correlation with either closely related stomata type (solid dots, *n* = 21, *p* = .189, Student's *t*‐test) or distantly related stomata (open dots, *n* = 34, *p* = 0.122, Student's *t*‐test).

One important characteristic of clustered stomata is the short distance separating individual stomata. To assess whether stomatal dynamics are influenced by neighboring stomata, we calculated the mean distance (X̅) between one stomate and its close neighbors in *B. formosana* and *B. hernandioides* (Figure 3a and b). We obtained a weak but significant positive relationship between the rate of pore opening and X̅ in *B. hernandioides*, but not in *B. formosana* (*r* ^2^ = .22, *p* = .002, Figure 3b). Thus, the farther one stomate was from its neighbors, the faster it opened in the range of 40–120 μm in *B. hernandioides*. With an average width of 30 μm (this study) for clustered stomata, one stomate may thus compete with its nearest neighbors while opening (Edwards et al., 1976; Harrison et al., 2020). Therefore, although clustered stomata are synchronized in their opening, the degree of opening also reflected the topology of the cluster.

### 3.7 The largest stomate in a cluster facilitates stomatal dynamics of its closely related neighbors

The rate of pore opening was positively correlated with mean maximal stomatal size (Figure 2a), which prompted us to test whether bigger stomata exert a stronger influence on pore opening than smaller stomata within a cluster. We ranked stomata according to their final pore area in each cluster complex (Figure 3c and d, largest stomate being 1). We considered two types of interaction: closely related and distantly related to its neighbors (Figure 3c and d). When we plotted the rate of pore opening as a function of distance between closely related or distantly related neighboring stomata for the largest stomate, we observed a positive correlation for closely related stomata (Figure 3e). We followed a similar approach for neighboring stomata of a stomate with the smallest pore size. However, in this case we detected no relationship between the rate of pore opening and distance, regardless of a closely or distantly related interaction (Figure 3f). We conclude that the stomate with the largest pore size within a cluster plays a dominant role in facilitating stomatal dynamics for the entire stomatal cluster.

### 3.8 Begonia TMMs differentially rescue the *Arabidopsis tmm* mutant phenotype

**FIGURE 4:**
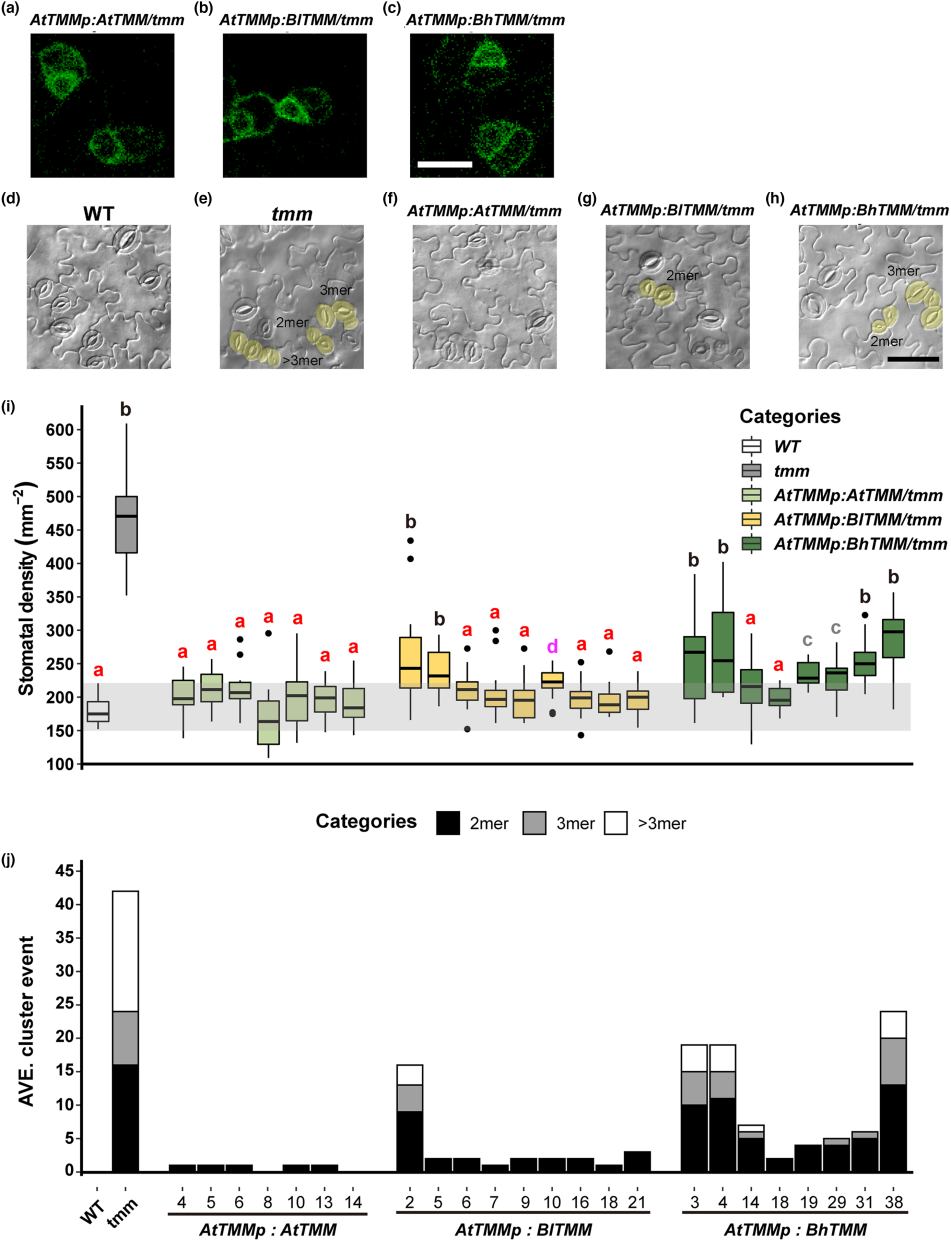
*Begonia TMM* expression patterns and their complementation of the *Arabidopsis tmm* mutant. (a–c) Fluorescence images in transgenic lines expressing *AtTMMp:AtTMM‐eYFP* (a), *AtTMMp:BlTMM‐eYFP* (b), and *AtTMMp:BhTMM‐eYFP* (c) in the *tmm* knockout background. *Begonia* TMMs localize to the plasma membrane as *Arabidopsis* TMM. Scale bar, 10 μm. (d–h) Differential interference contrast (DIC) images of cotyledon epidermis from Col‐0 (wild type, WT) non‐transgenic control (d), *tmm* (e), *AtTMMp:AtTMM‐eYFP* (f), *AtTMMp:BlTMM‐eYFP* (g), and *AtTMMp:BhTMM‐eYFP* (h) seedlings. The categories of cluster events are indicated: stomatal pairs, 2‐mer; three clustered stomata, 3‐mer; more than three adjacent stomata, >3‐mer. Clusters of stomata are highlighted in yellow. Scale bar, 50 μm. (i and j) Stomatal density (SD) (i) and average number of cluster events (j) in *Arabidopsis tmm* complementation lines transformed with *Arabidopsis* or *Begonia TMM*. T_2_ lines with positive TMM‐eYFP signals were used for quantification. The WT (Col‐0) and the *tmm* knockout mutant were used as positive and negative controls, respectively. The gray area represents the SD range in the WT (*n* = 13 for the WT and *tmm*; *n* = 5–20 for complementation lines). Different lowercase letters indicate significant differences, *p* < .05, Kruskal‐Wallis one‐way ANOVA on ranks and Dunn's test.

How are stomatal clusters formed? *TMM*, a single‐copy and well‐conserved gene, encodes a membrane receptor that negatively regulates stomatal formation in many plant species (Peterson et al., 2010). Loss of TMM function resulted in contiguous stomatal clusters that restrict CO_2_ diffusion and reduce the photosynthetic potential of a leaf (Dow et al., 2014; Lawson & Blatt, 2014). As the *B. hernandioides* or *B. formosana* genome sequences are not available, we searched the draft genome of *B. luzhaiensis* (with solitary stomata) with *Arabidopsis TMM* as a query. We identified one *TMM* sequence from *B. luzhaiensis*. As *B. formosana* is an allopolyploid, we used *B. luzhaiensis* as another example of a *Begonia* species with solitary stomata. With the primers designed from *B. luzhaiensis TMM* genomic sequences, we cloned and sequenced *TMM* from *B. hernandioides*. The encoded TMM proteins from *B. hernandioides* (BhTMM; clustered stomata) and *B. luzhaiensis* (BlTMM; solitary stomata) were longer than their *Arabidopsis* ortholog (521 amino acids for *Begonia* and 496 amino acids for *Arabidopsis*) (Figure S2). Based on sequence predictions of protein families and domains (Prosite) (Sigrist et al, 2012), both *Begonia* TMMs had several leucine‐rich repeat (LRR) domains near the C terminus, like *Arabidopsis* TMM. The most obvious difference between *Begonia* and *Arabidopsis* TMM was the addition of 38 amino acids with an unknown function at the N terminus of *Begonia* TMM. We also compared *Begonia* TMMs with other TMM protein sequences obtained from the 1000 Plants (1KP) project (Leebens‐Mack et al., 2019) and the website of the Universal Protein Resource (UniProt) (The UniProt Consortium, 2021). The phylogenetic tree indicated that TMM is conserved among families in eudicots (Figure S3). To explore the function of *Begonia* TMMs, we cloned the *Begonia TMM* genes downstream of the *Arabidopsis AtTMM* promoter and in‐frame of the enhanced yellow fluorescent protein (*eYFP*) sequence and introduced the resulting constructs in the *Arabidopsis tmm* mutant. All three TMM constructs (*AtTMM*, *BhTMM*, and *BlTMM*) showed YFP fluorescence associated with the plasma membrane in the stomatal lineage (Figure 4a–c). In addition, *AtTMM* and the two *Begonia TMM* constructs rescued normal stomatal clustering in the *tmm* mutant (Figure 4d–h) and returned stomatal density from ca. 469 mm^−2^ in *tmm* to wild‐type levels (on average ca. 225 mm^−2^ for *AtTMM*, 212 mm^−2^ for *BlTMM*, and 247 mm^−2^ for *BhTMM*) (Figure 4i). The stomatal clusters were smaller in all complementation lines compared to the clusters observed in the *tmm* mutant, suggesting that *Begonia* TMMs can prevent cluster formation as Arabidopsis TMM does (Figure 4j). However, the expression of *BlTMM* or *BhTMM* in the *tmm* mutant did not fully rescue the mutant phenotypes in terms of stomatal density (Figure 4i) and stomatal cluster events (Figure 4j). In summary, the localization and function of *Begonia* TMMs were similar to that of AtTMM, but BhTMM from a species with clustered stomata was less efficient in reducing stomatal density and clustering in *tmm* mutants than *BlTMM* from a species with solitary stomata.

## 4 DISCUSSION

Stomatal clustering and multiseriate (multilayer) hypodermis in *Begonia* appear to be correlated with drought tolerance in field studies (Gan et al., 2010; Rudall et al., 2018). Mathematical modeling suggested that the transpiration rate (*E*) could be reduced by 5–15% through overlapping vapor shells in clustered stomata (Lehmann & Or, 2015), which improved WUE if the same assimilation rate (*A*) was maintained. Consistent with the reports, our observation in Figure 1h suggested that the increased WUEi and instantaneous WUE at 100–200 μmol·m^−2^ s^−1^ were due to the improved photosynthetic potential (*A*) and the reduced stomatal conductance (g_s_) in *B. hernandioides*. *B. hernandioides* (with clustered stomata) was originally collected from a rocky environment where it experiences partial water deficit, while *B. formosana* (with solitary stomata) was collected from the understory soil layer and does not suffer from water shortage. Although these two *Begonia* species have been grown in the same environment (greenhouse) for many years, their habitats and differing WUE suggested a connection between environmental adaptation and genetic control of stomata development.

### 4.1 Leaf anatomy and WUEi

*B. hernandioides* exhibited thicker leaves and higher leaf mass per area (LMA) than *B. formosana* (Tables 1). The study of evergreen tree species in Japanese warm temperate forests showed that leaves with thicker mesophyll tended to have higher LMA, larger surface aera of mesophyll cells exposed to intercellular air spaces, and higher carbon isotopic composition in leaf dry mass (Hanba et al., 1999). Because carbon isotopic composition was correlated with water‐use efficiency in crops, it suggested that higher leaf mass per area is correlated to higher WUE (Hanba et al., 1999). The well‐developed multiseriate hypodermis beneath both epidermis layers in *B. hernandioides* (Figure 1 and Table 1) provides a large substomatal cavity to increase carbon capture (Roth‐Nebelsick, 2007) that would also optimize the placement of photosynthetic mesophyll cells (Rudall et al., 2018). In woody angiosperm species, LMA is positively related to the sensitivity of stomatal closure during dehydration (Henry et al., 2019). Therefore, *B. hernandioides* with a large substomatal cavity and higher LMA may facilitate the gas exchange and be more sensitive to dehydration stress to survive in the arid environment.

### 4.2 Stomatal morphology and dynamics

The anatomical features of stomata, such as stomatal number and size, are strongly correlated to *g*_*s*_ (Lawson & Vialet‐Chabrand, 2019). We expected plants with higher stomatal density due to clustering to have lower *A* (Dow et al., 2014). However, we observed that *B. hernandioides* instead displays a lower stomatal density compared to *B. formosana* (Table 2), suggesting coordination between stomatal morphology and density to achieve a proper balance between stomatal number and photosynthesis efficiency. *g*_*s max*_, the theoretical maximum conductance assuming fully open stomata under ideal conditions (Conesa et al., 2019), is calculated using the theoretical maximum stomatal pore (*a*_*max*_), stomatal density, and stomatal depth (l) (Dow et al., 2017; Harrison et al., 2020). Although we did measure a slight difference for *g*_*s*_ between the two *Begonia* species (Table 2), *g*_*s max*_ was significantly lower in *B. hernandioides* relative to *B. formosana*. In combination with our other observations, the difference between *g*_*s*_ and anatomical *g*_*s max*_ suggested that the degree of stomatal opening is different in these two *Begonia* species.

Small stomatal size (guard cells) is correlated with a fast response to the environment (Drake et al., 2013; Henry et al., 2019; Papanatsiou et al., 2017). In our study, the clustered stomata of *B. hernandioides* were smaller (Table 2) and showed a faster opening response following the transition from darkness to light when compared to the solitary stomata of *B. formosana* (30–90 min Figure 2b). Moreover, stomatal opening in *B. hernandioides* behaved more synchronously upon exposure to light, reaching half pore opening faster than in *B. formosana* at 90–120 min (Figure 2c) and with more stomata bearing a bigger pore size at 120 min (Figure 2d). Although evidence is currently lacking in the literature to support whether the synchronicity in stomatal response is beneficial to carbon gain or/and water loss, synchronous stomatal movements were reported in black elder (*Sambucus nigra*), a species with solitary stomata (Kaiser & Kappen, 2001). We speculate that the combination of fast stomatal opening and synchronicity may maximize carbon uptake, resulting in higher photosynthetic potential in *B. hernandioides*.

A specific communication between stomata within a cluster, such as mechanical forces through turgor pressure from surrounding epidermal cells or signal transduction from internal leaf tissue, may facilitate or synchronize stomatal movement. Improper spacing between stomata may impede ion exchange between cells, resulting in stomatal misfunction (Kim et al., 2010; Outlaw Jr., 1983). For example, *Arabidopsis tmm* mutant plants violate the single‐cell spacing rule, as it is characterized by contiguous clustered stomata, which reduces the accumulation of potassium (K^+^) ions and K^+^ channel activity in guard cells, leading to impaired guard cell function (Papanatsiou et al., 2016). Subsidiary cells adjacent to guard cells may facilitate guard cell movement by offering a reservoir for water and ions, and modify stomatal morphologies (i.e., sunken and raised stomata) to improve gas exchange (Gray et al., 2020). In monocots, subsidiary cells have been reported to control stomatal kinetics. Franks and Farquhar (2007) reported that maximum stomatal opening cannot be achieved without a reduction in the turgor pressure of subsidiary cells. Moreover, the loss of subsidiary cells in *Brachypodium distachyon* negatively affected stomatal kinetics and yielded lower *g*_*s*_ (Raissig et al., 2017). These observations underscore the role of subsidiary cells in stomatal opening. *B. hernandioides* has non‐contiguous stomatal clusters with cells spaced in a cluster (Figure 1), and the farther one stomate was from its neighbors, the faster it opened in the range of 40–120 μm within a cluster (Figure 3b). It is possible that the arrangement between epidermal cells could limit each other by influencing the influx and efflux of solutes (Drake et al., 2013; Hetherington & Woodward, 2003) or by interfering with the physical tension on the surface, thereby modulating the stomatal behaviors in a cluster. Moreover, stomatal kinetics have been reported to respond to vapor pressure deficit (Merilo et al., 2018) and internal CO_2_ concentrations (*C*_*i*_) (Engineer et al., 2016) through internal signal transduction cascades. We cannot exclude these factors as contributing to the results in our experiments, as we used the whole tissue of leaf. Therefore, the coordination between stomata in species with clustered stomata may reflect the mechanical force caused by ionic exchange between each stomate as well as the signal(s) derived from the internal leaf cell layers.

### 4.3 Begonia TMMs

*Arabidopsis* TMM plays an important role in spacing stomata over the surface of the leaf epidermis (Yang & Sack, 1995). Loss of TMM function causes contiguous stomatal clustering, making *Begonia* TMM a good candidate responsible for variation in cluster formation. However, *TMM* from either *Begonia* species with solitary or clustered stomata rescued the *Arabidopsis tmm* mutant in terms of stomatal density (Figure 4). Notably, *BhTMM* from the species with clustered stomata did not complement the *tmm* mutant to the same extent as *BlTMM* from the species with solitary stomata (Figure 4i and j), suggesting that the canonical role of TMM is to mediate cell‐to‐cell signaling. By contrast, the magnitude of inhibition of stomatal development may vary among *Begonia* TMMs, with TMM from species with clustered stomata being less active than TMM from solitary stomata, raising the possibility that the efficacy of TMM may be one of the factors contributing to the formation of clustered stomata. Future studies of isolating and comparing TMM sequences from genetically related *Begonias* will reveal the importance of TMM in forming clustered stomata.

In conclusion, the xerophytic species *Begonia* with stomatal clusters showed higher WUEi due to its small and more closely packed stomata. The rapid and synchronous stomatal movements within a cluster ensured efficient CO_2_ intake, while the multiseriate hypodermis also provides a reservoir of carbon for photosynthesis. TMM from *Begonia* species with clustered stomata may be less active compared to species with solitary species, providing a potential rationale for the observed clustering. Therefore, besides manipulating the number of stomata, stomatal clustering may offer an additional developmental strategy by which to modulate leaf structure, stomatal morphology, and dynamics to achieve better WUEi. Further exploration of the molecular mechanisms behind stomatal clustering and the formation of a multiseriate hypodermis in a leaf using different *Begonia* species will advance our knowledge of plant fitness, adaptation, and evolution.

## AUTHOR CONTRIBUTIONS

MYT, CK, HAY, KFC, and CMKH designed experiments. MYT, CK, and ZLG performed the wet‐lab and statistical analysis. HAY and KFC provided and maintained plant materials. MYT, KFC, and CMKH wrote the manuscript.

## CONFLICT OF INTEREST

The authors declare no competing interests.

## Supporting information


Appendix S1

## Data Availability

The data available in the article supplementary.
