# Supplementary material for: Stomatal clustering in Begonia improves water use efficiency by modulating stomatal movement and leaf structure
Source: Plant Environ Interact. 2022 Jul 4;3(4):141–54. doi: 10.1002/pei3.10086 (PMC10168073; doi:10.1002/pei3.10086)
Supplement: Supplementary file 1 — Appendix S1 [file PEI3-3-141-s001.pdf]

Supplemental materials for

Stomatal clustering in *Begonia* improves water use efficiency by modulating stomatal movement and leaf structure

Meng-Ying Tsai[1,2], Chi Kuan[1], Zheng-Lin Guo[1], Hsun-An Yang[2], Kuo-Fang Chung[2*], Chin-Min Kimmy Ho[1*]

[1] Institute of Plant and Microbial Biology, Academia Sinica, Taipei, Taiwan

[2] Research Museum and Herbarium (HAST), Biodiversity Research Center, Academia Sinica, Taipei, Taiwan

*Author for correspondence

This PDF file includes

Supplemental Figure 1 to 3

Supplemental Table 1

Supplemental References

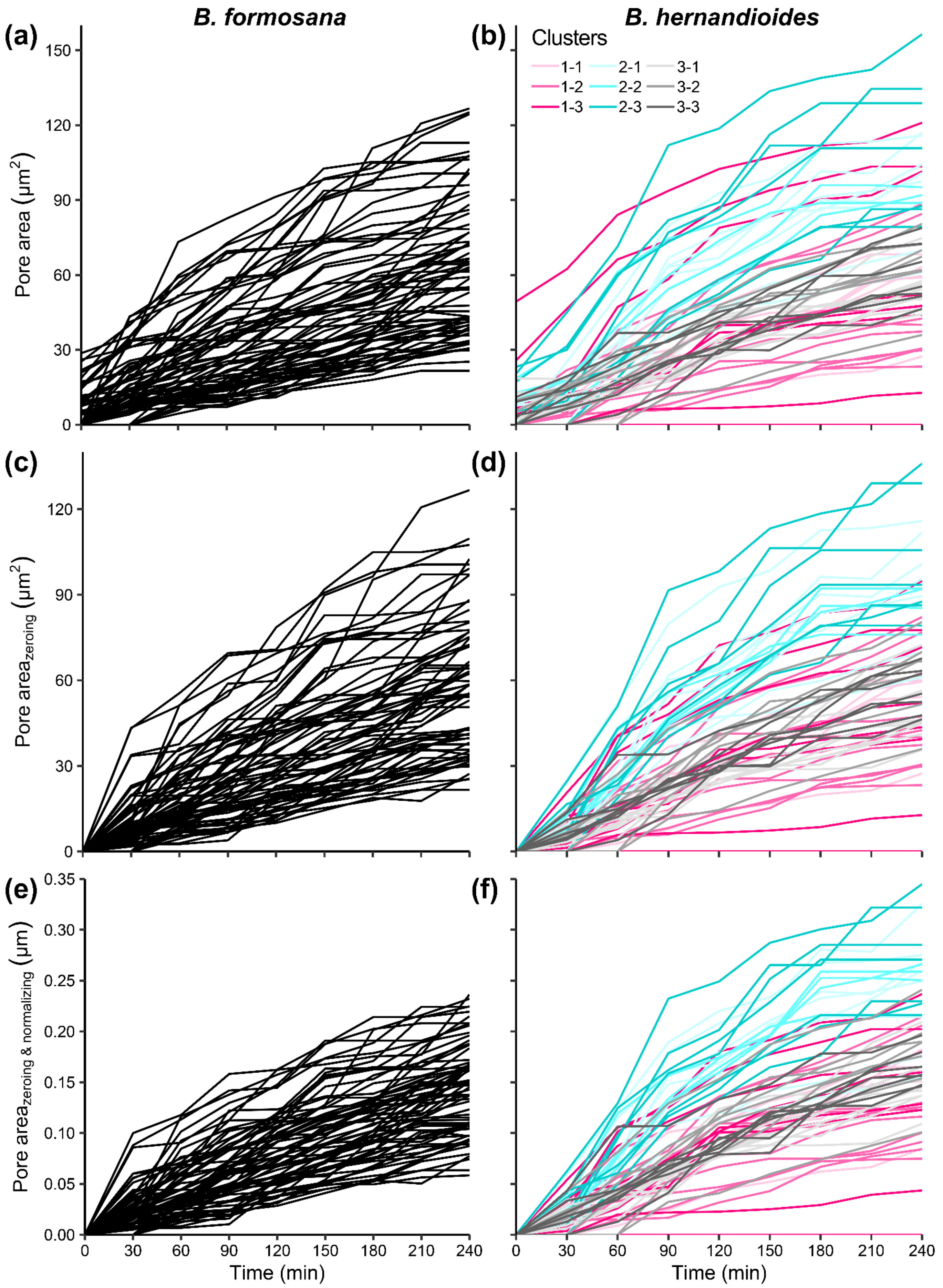

B. formosana
B. hernandioides
(a)
(b)
(c)
(d)
(e)
(f)
Clusters
1-1
1-2
1-3
2-1
2-2
2-3
3-1
3-2
3-3
Pore area (μm2)
Pore area zeroing (μm2)
Pore area zeroing & normalizing (μm)
Time (min)
0
30
60
90
120
150
180
210
240
0.00
0.05
0.10
0.15
0.20
0.25
0.30
0.35

(g)

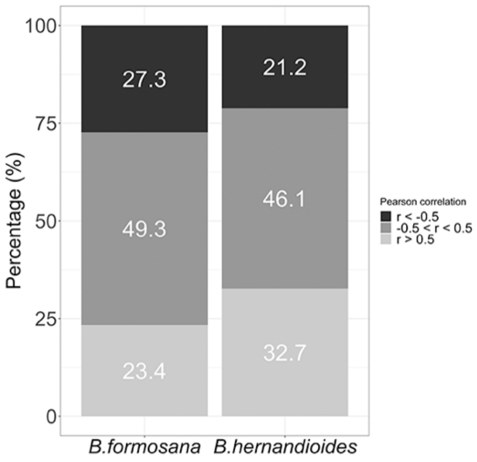

Figure S1. Response of pore area from 0 to 240 min under an irradiance of 100–200 μmol·m^{-2}·s^{-1} in opening buffer.

(*a* and *b*) Raw data from changes in pore area from 0 to 240 min under an irradiance of 100–200 μmol·m^{-2}·s^{-1} in *B. formosana* (*a*) and *B. hernandioides* (*b*).

(*c* and *d*) Pore area subtracted by the pore area at the starting point (0 min) (zeroing) in *B. formosana* (*c*) and *B. hernandioides* (*d*).

(*e* and *f*) Normalized data after zeroing and normalization by the maximum length of each stomate in *B. formosana* (*e*) and *B. hernandioides* (*f*).

(g) Pearson's correlation coefficients (*r*) for pairwise comparisons between individual opening dynamics from 30 to 150 minutes using three individuals form *B. formosana* and individuals #1 and #3 from *B. hernandioides*. There were more positive correlations in *B. hernandioides* than in *B. formosana*.

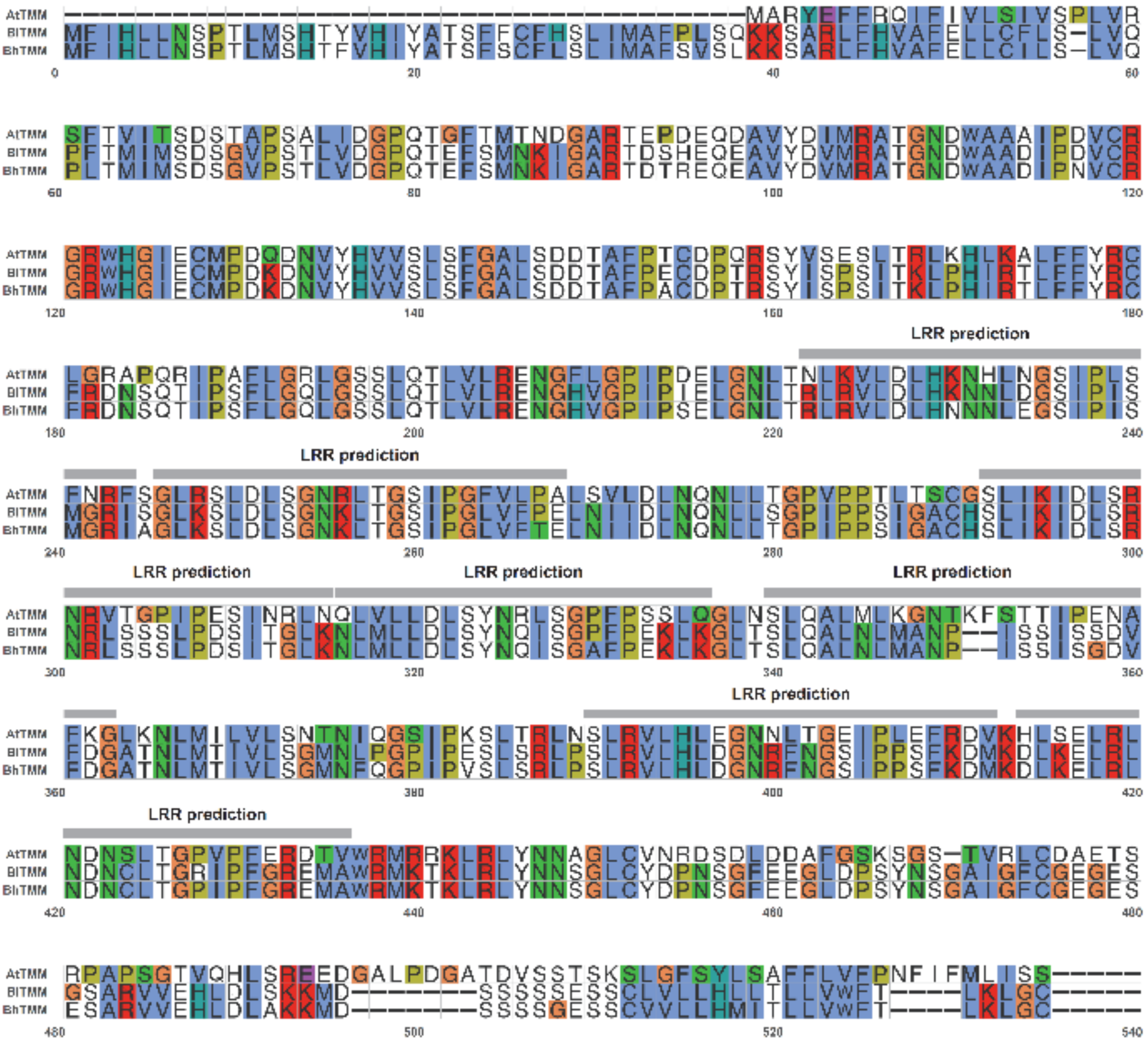

Figure S2. Amino acid alignment of AtTMM, BlTMM, and BhTMM.

Begonia TMM (521 amino acids) is longer than *Arabidopsis* TMM (496 amino acids). The alignments were performed with the CRAN Package *ggmsa* (https://CRAN.R-project.org/package=ggmsa) in R version 4.0.3. Color patches were based on the ClustalX color scheme. Leucine-rich repeat (LRR) domains were predicted using the PROSITE database (50).

7

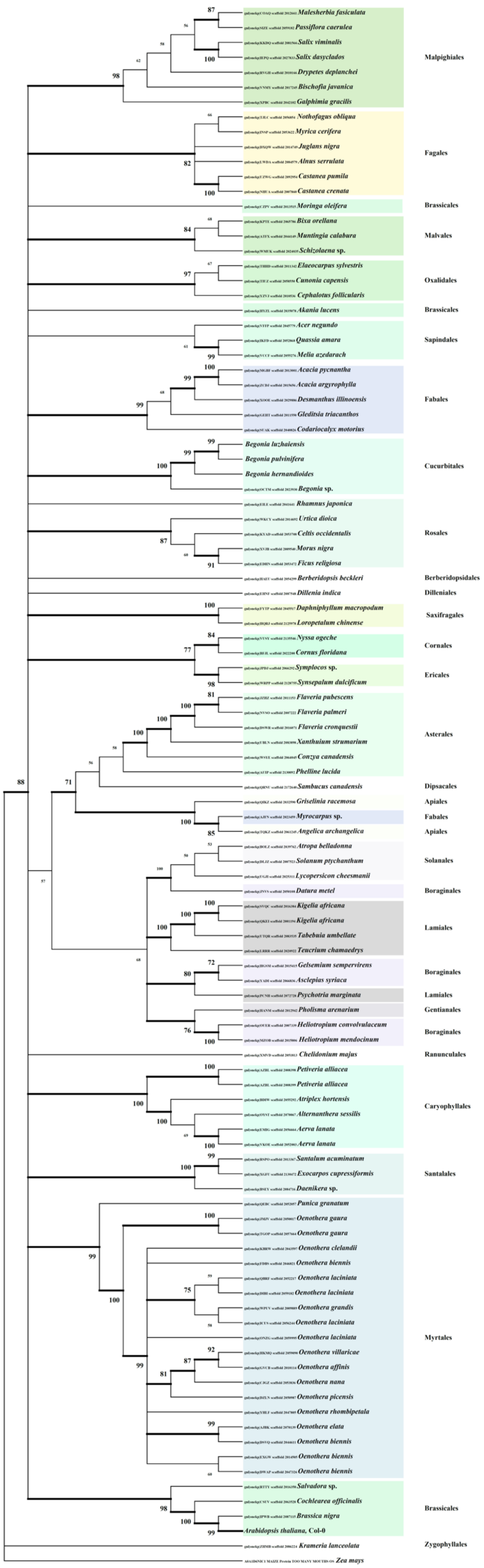

Malpighiales
Fagales
Brassicales
Malvales
Oxalidales
Brassicales
Sapindales
Fabales
Cucurbitales
Rosales
Berberidopsidales
Dilleniales
Saxifragales
Cornales
Ericales
Asterales
Dipsacales
Apiales
Fabales
Apiales
Solanales
Boraginales
Lamiales
Boraginales
Lamiales
Gentianales
Boraginales
Ranunculales
Caryophyllales
Santalales
Myrtales
Brassicales
Zygophyllales

Figure S3. The optimal tree of TMMs was shown using the Neighbor-Joining method (Saitou & Nei, 1987). This analysis involved 105 amino acid sequences. The percentage of replicate trees in which the associated taxa clustered together in the bootstrap test (1000 replicates) are shown next to the branches (Felsenstein, 1985).For Condensed tree, Cut-off value is set to BS = 50%. Bootstrap Support (BS) ≥ 70% were shown in bold types and lines. Most TMMs with strong support (BS> 70 %) in order-level represented similar phylogenetic relationships within orders; however, phylogenetic relationships among orders could not be confidently resolved with weak support (BS<70%). The evolutionary distances were computed using the JTT matrix-based method (Jones *et al.*, 1992) and are in the units of the number of amino acid substitutions per site. All positions with less than 50% site coverage were eliminated (partial deletion option). There were a total of 455 positions in the final dataset. Evolutionary analyses were conducted in MEGA X (Kumar *et al.*, 2018).

Table S1. Parameters of gas exchange.

Net CO_2 assimilation rate (*A*, µmol m^{-2} s^{-1}), stomatal conductance (*gs*, mol H_2O m^{-2} s^{-1}), and transpiration rate (*E*, mmol H_2O m^{-2} s^{-1}) under three light intensities in *B. formosana* (solitary stomata) and *B. hernandioides* (clustered stomata) (*n* = 3 biological replicates). No significant differences were detected between the two *Begonia* species, as determined by Student's *t*-test.

Parameters	PPFD (µmol m^{-2} s^{-1})	Species	
		B. formosana	*B. hernandioides*
A (µmol m^{-2} s^{-1})	25	1.41 ± 0.25	1.35 ± 0.17
	100	2.93 ± 0.54	3.02 ± 0.35
	200	3.19 ± 0.61	3.96 ± 0.81
g_s (mol H_2O m^{-2} s^{-1})	25	0.05 ± 0.02	0.03 ± 0.02
	100	0.04 ± 0.01	0.03 ± 0.00
	200	0.04 ± 0.01	0.04 ± 0.01
E (mmol H_2O m^{-2} s^{-1})	25	0.52 ± 0.16	0.30 ± 0.18
	100	0.42 ± 0.08	0.29 ± 0.05
	200	0.45 ± 0.11	0.41 ± 0.13

Felsenstein J. 1985. Confidence limits on phylogenies: an approach using the bootstrap. *evolution* **39**(4): 783-791.

Jones DT, Taylor WR, Thornton JM. 1992. The rapid generation of mutation data matrices from protein sequences. *Bioinformatics* **8**(3): 275-282.

Kumar S, Stecher G, Li M, Knyaz C, Tamura K. 2018. MEGA X: molecular evolutionary genetics analysis across computing platforms. *Molecular biology and evolution* **35**(6): 1547.

Saitou N, Nei M. 1987. The neighbor-joining method: a new method for reconstructing phylogenetic trees. *Molecular biology and evolution* **4**(4): 406-425.
